# Cyclooxygenase metabolism mediates vasorelaxation to 2-arachidonoylglycerol (2-AG) in human mesenteric arteries

**DOI:** 10.1016/j.phrs.2014.02.001

**Published:** 2014-03

**Authors:** Christopher P. Stanley, Saoirse E. O'Sullivan

**Affiliations:** School of Medicine, University of Nottingham, Royal Derby Hospital, Derby DE22 3DT, United Kingdom

**Keywords:** 2-AG, 2-arachidonoylglcerol, AEA, anandamide, ANOVA, analysis of variance, CB_1_, cannabinoid receptor one, CB_2_, cannabinoid receptor two, COX-1, cyclooxygenase one, COX-2, cyclooxygenase two, ECS, endocannabinoid system, FAAH, fatty acid amide hydrolase, KPSS, high potassium physiological saline solution, l-NAME, *N*^G^-nitro-l-arginine methyl ester, MAGL, monoacylglycerol lipase, PPAR, peroxisome proliferator activated receptor, PSS, physiological saline solution, SEM, standard error of mean, TRPV1, transient receptor potential vanilloid-1, Endocannabinoid, 2-Arachidonoylglycerol, Vasorelaxation, Cyclooxygenase, Prostanoid, Human

## Abstract

**Objective:**

The vasorelaxant effect of 2-arachidonoylglycerol (2-AG) has been well characterised in animals. 2-AG is present in human vascular cells and is up-regulated in cardiovascular pathophysiology. However, the acute vascular actions of 2-AG have not been explored in humans.

**Approach:**

Mesenteric arteries were obtained from patients receiving colorectal surgery and mounted on a myograph. Arteries were contracted and 2-AG concentration–response curves were carried out. Mechanisms of action were characterised pharmacologically. *Post hoc* analysis was carried out to assess the effects of cardiovascular disease/risk factors on 2-AG responses.

**Results:**

2-AG caused vasorelaxation of human mesenteric arteries, independent of cannabinoid receptor or transient receptor potential vanilloid-1 activation, the endothelium, nitric oxide or metabolism *via* monoacyglycerol lipase or fatty acid amide hydrolase. 2-AG-induced vasorelaxation was reduced in the presence of indomethacin and flurbiprofen, suggesting a role for cyclooxygenase metabolism 2-AG. Responses to 2-AG were also reduced in the presence of Cay10441, L-161982 and potentiated in the presence of AH6809, suggesting that metabolism of 2-AG produces both vasorelaxant and vasoconstrictor prostanoids. Finally, 2-AG-induced vasorelaxation was dependent on potassium efflux and the presence of extracellular calcium.

**Conclusions:**

We have shown for the first time that 2-AG causes vasorelaxation of human mesenteric arteries. Vasorelaxation is dependent on COX metabolism, activation of prostanoid receptors (EP_4_ & IP) and ion channel modulation. 2-AG responses are blunted in patients with cardiovascular risk factors.

## Graphical abstract

**Figure:**
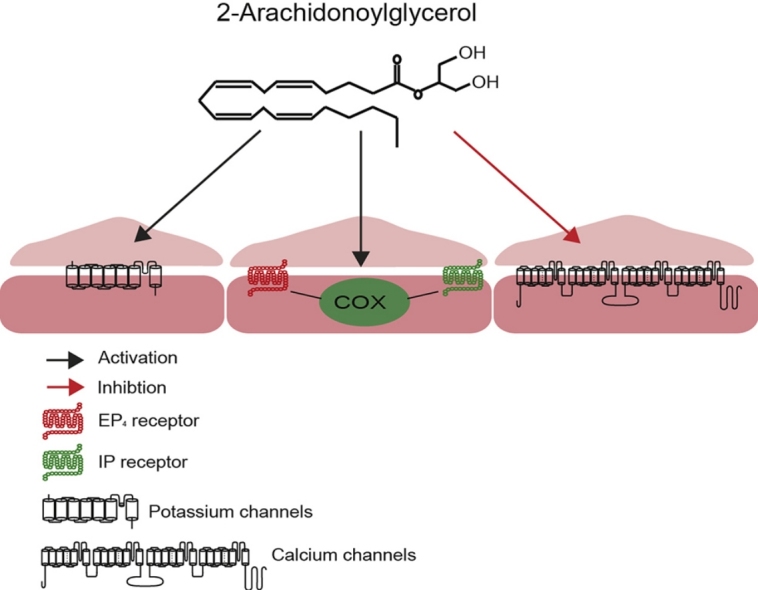

## 1 Introduction

The endocannabinoid 2-arachidonoylglycerol (2-AG), found in human patient plasma at low nanomolar concentrations [1], functions in a paracrine manner [2]. Synthesis of 2-AG occurs on demand through hydrolysis of a range of phospholipid precursors, and degradation occurs through multiple pathways including monoacylglycerol lipase (MAGL), fatty acid amide hydrolase (FAAH) and cyclooxygenase (COX) dependent pathways [3]. 2-AG binds to the CB_1_ and CB_2_ receptors at high nanomolar and low micromolar concentrations respectively [4], and causes slight calcium influx through TRPV1 channels [5].

*In vivo*, 2-AG administration induces hypotension through COX metabolism in mice [6], and through the CB_1_ receptor in rats [7]. *In vitro*, 2-AG causes vasorelaxation of bovine coronary arteries that is dependent on metabolism by MAGL, FAAH, COX and cytochrome P450 [8]. Conversely, in rat mesenteric arteries, metabolism of 2-AG appears to limit vasorelaxation [9] and causes contraction of the aorta [10]. In rabbit mesenteric arteries, 2-AG-induced vasorelaxation is unaffected by inhibition of COX and nitric oxide, blockage of potassium channels or endothelium denudation, but is inhibited by high concentrations of CB_1_ antagonist [11]. Variations between these results may be accredited to the different species used or different vascular beds studied [12]. To date, no studies have examined the vascular responses of 2-AG in humans.

Levels of 2-AG, and other components of the endocannabinoid system, have been shown to be altered in human vascular disease and models of vascular disease including atherosclerosis [13,14] and heart disease [15,16]. A full understanding as to the reasons for these alterations in cardiovascular pathophysiology is as yet unclear. However, there is evidence to suggest that in some forms of cardiovascular dysfunction, increased endocannabinoid activity may be protective. Endocannabinoid stimulation of cannabinoid receptors causes greater vasorelaxation in hypertensive rats compared to normotensive counterparts [17], decreases contractile sensitivity to the thromboxane mimetic U46619 in rat middle cerebral arteries [18], reduces myocardial infarct size [19,20], reduces atherosclerotic plaque size [21] and reduces cardiac ischaemia/reperfusion injury [22].

Despite the fact that 2-AG and other components of the endocannabinoid system are present in the human cardiovascular system [23–25,15] and that 2-AG causes release of nitric oxide from human endothelial cells in a CB_1_ dependant manner [26] the acute vascular effects of 2-AG have not been studied in human arteries. Understanding the pharmacological effects of this endogenous cannabinoid in human vascular tissue may aid in further understanding of any potential role of 2-AG and the endocannabinoid system in human cardiovascular pathologies. Therefore, the aim of this study was to examine the acute vascular effects of the 2-AG and characterise the underlying pharmacology in human mesenteric arteries.

## 2 Methods

### 2.1 General methods

The Derbyshire Research Ethics Committee and Derbyshire Hospitals Trust Research and Development granted ethical approval for the use of mesenteric tissue from patients undergoing surgical treatment of bowel carcinoma and inflammatory bowel disorders (patient characteristics supplementary data Table 1). Excised mesenteric tissue was placed in physiological saline solution (PSS) (composition, mmol/L: Sodium chloride 119, potassium chloride 4.7, calcium chloride 2.5, magnesium sulfate 1.17, sodium bicarbonate 25, potassium phosphate 1.18, EDTA 0.027, glucose 5.5, dissolved in triple distilled water) and transported back to the lab. Arteries (668 ± 40 μm S.E.M diameter, *n* = 162 arterial segments from 44 patients) were dissected from mesenteric tissue, cleaned of adherent adipose and connective tissue and cut into 2 mm segments. Artery segments were mounted on fine tungsten wires (40 μm diameter) on a myograph (Danish Myo Technology, Denmark) at 37 °C in PSS solution and gassed with 5% CO_2_ in O_2_. Tension was measured using isometric force displacement transducers and recorded using Chart 5 Pro (ADInstruments, Oxfordshire, UK). Once mounted, arteries were normalised using normalisation software (ADInstruments, Oxfordshire, UK), setting the arteries to an internal diameter that produced 90% of 13.3 kPa (0.9L_100_) [27], this gave an average baseline of 4.2 ± 0.2 mN (*n* = 162 arterial segments from 44 patients). Artery segments were either used fresh (*n* = 24) or after overnight storage in PSS at 4 °C (*n* = 20). Initial experiments revealed overnight storage had no significant effect on the contractile or relaxation responses of mesenteric arteries (supplementary data Fig. 1A–D). Arteries were contracted using high potassium physiological saline solution (124 nmol/L KPSS) (composition, mmol/L: Sodium chloride 0, potassium chloride 124, calcium chloride 2.5, magnesium sulfate 1.17, sodium bicarbonate 25, potassium phosphate 1.18, EDTA 0.027, glucose 5.5 all dissolved in triple distilled water) and 50 nmol/L U46619, and were relaxed using 10 μmol/L bradykinin. An increase in tension of greater than 5 mN to both KPSS (20.3 ± 0.7 mN increase in tone, *n* = 162 arterial segments from 44 patients) and U46619 (78 ± 2.1% second KPSS response, *n* = 162 arterial segments from 44 patients), and a relaxation greater than 70% to bradykinin (mean 88 ± 1.5% relaxation, *n* = 162 arterial segments from 44 patients) against U46619 pre-imposed tone was used to validate smooth muscle and endothelium function [28].

### 2.2 Experimental protocol

Viable arteries were contracted using combinations of U46619 (50–250 nmol/L: and endothelin-1 (1–3 nmol/L). Once a stable contraction greater than 5 mN (mean 16 ± 0.6 mN, *n* = 162 arterial segments from 44 patients) was achieved, cumulative concentration–response curves were constructed to 2-AG. 2-AG was added in 5-min intervals with measurements taken, and expressed as percentage relaxation of pre-imposed tone, in the final minute of each concentration addition. Responses were compared to ethanol-treated vehicle controls carried out in adjacent arterial segments from the same patient. In experiments to determine the effects of 2-AG on baseline tone, arteries were not contracted and a 2-AG concentration-response curve performed on arteries at normalised baseline tone. Any response was then subtracted off the pre-2-AG baseline and expressed as Δ alterations in tone, this was compared to vehicle treated control segments of adjacent arteries from the same patient. All arteries used in this study were taken from the mesentery distal to the colon smooth muscle wall. However, given that the operation type (right hemicolectomy, left hemicolectomy, anterior resection/abdominoperineal resection and total colectomy) may influence vascular responses based on different blood supply from differing feed vessels, 2-AG concentration response curves were compared to reveal no significant difference in the 2-AG concentration response curve in arteries obtained through different surgeries (supplementary data Fig. 1E). In subsequent experiments to characterise the mechanisms that underpin 2-AG-induced vasorelaxation, the patient's samples were randomly assigned to a range of intervention groups. The split of patients between intervention groups is shown in supplementary data Table 4, showing no distinct pattern that could potentially influence results. In intervention studies, a length of arterial branch was taken from the patient sample. This branch was then typically cut into four 2 mm segments, one segment was treated with 2-AG alone (control), the remaining three segments were pre-treated with a range of interventions (described below) and then treated with 2-AG.

Potential cannabinoid receptor involvement in 2-AG-induced vasorelaxation was assessed with the CB_1_ and cannabinoid receptor 2 (CB_2_) receptor antagonists AM251 (100 nmol/L) and AM630 (100 nmol/L) both incubated for 10 min prior to artery contraction [29,30]. Desensitisation of the TRPV1 receptor was accomplished *via* incubation (1 h) with the TRPV1 agonist, capsaicin (10 μmol/L) followed by 3 washouts in PSS [5]. In some experiments, the endothelium was removed by gentle abrasion using a human hair [30], removal of the endothelium was confirmed by a less than 10% vasorelaxation to 10 μmol/L bradykinin. A role for the involvement of nitric oxide was investigated using *N*^G^-nitro-l-arginine methyl ester (l-NAME, 300 μmol/L) present throughout [30].

Metabolism of 2-AG into vasoactive compounds was assessed by 10 min pre-contraction incubation with the MAGL inhibitor JZL184 (1 μmol/L) and the FAAH inhibitor URB597 (1 μmol/L) [9]. The potential role of COX metabolism was assessed using the non-selective COX inhibitor indomethacin (10 μmol/L) [31], the COX-1 favourable inhibitor flurbiprofen (10 μmol/L) or the COX-2 inhibitor nimesulide (10 μmol/L) [9] all present throughout. A role for vasorelaxant prostanoid receptors was investigated using; the DP_1_/EP_2_ receptor antagonist AH6809 (initially at a concentration of 10 μmol/L, however this significantly inhibited contractile responses therefore a concentration of 1 μmol/L was used, 30 min incubation) [32], the EP_4_ receptor antagonist L-161982 (1 μmol/L, 10 min prior to contraction) [33] or the IP receptor antagonist Cay10441 (100 nmol/L, 10 min incubation) [34].

A potential role for potassium channel hyperpolarisation was investigated by carrying out concentration–response curves to 2-AG in arteries contracted using KPSS to inhibit potassium efflux [35]. To investigate whether 2-AG causes vasorelaxation in a calcium-dependant manner, arteries were contracted using U46619 in calcium-free PSS. To investigate the effects of 2-AG on calcium influx, concentration–response contractions to calcium chloride were carried out in arteries that were incubated with calcium-free PSS (10 min), calcium-free KPSS (10 min) and then 2-AG (10 min with 10 or 100 μmol/L) or vehicle control (1% ethanol) [35].

The effect of antagonists/inhibitor incubations on baseline tone can be seen in supplementary data Table 3. It should be noted that capsaicin pre-treatment significantly lowered baseline tone and incubation with 10 μmol/L AH6809 significantly inhibited U46619 and endothelin-1 contraction. All other techniques employed did not affect arterial contraction compared to arteries that had no prior incubation.

### 2.3 Statistical analysis

Graphs represent mean percentage relaxations, with error bars representing the standard error of the mean (S.E.M.), and *n* equalling the number of patients unless otherwise stated. Sigmoidal concentration–responses curves with a standard hill slope of 1 were fitted to those data (Prism Version 5; GraphPad Software, California, USA). In vasorelaxation studies comparisons were made between intervention and control artery segments from the same patient. Despite the clear alterations in 2-AG concentration response curves in the presence of a range of inhibitors and antagonists, the effects on pEC_50_ and *R*_max_ values were modest (supplementary data Table 2). Therefore, data were also analysed using paired Student *t*-tests of area under the curve (AUC) to detect overall alterations in concentration response curves. The AUC was defined as the area between the mean response value and the baseline value of 0% relaxation (100% of contractile tone). In CaCl_2_ contraction studies, comparisons of AUC were made using one-way analysis of variance (ANOVA) with Dunnetts *post hoc* analysis. Comparisons between responses to KPSS, U46619 and bradykinin in fresh tissue and tissue that had been stored overnight (supplementary data Fig. 1) or between gender based responses to 10 μmol/L bradykinin (supplementary data Fig. 2C) were made using Student's unpaired *t*-test. Comparisons of 2-AG response curves based on gender were made using Student's unpaired *t*-test of area under the curve (supplement data Fig. 2a). All correlations studies were made using linear regression. Significance was determined with *P* < 0.05.

### 2.4 Drugs

All salts, L-NAME, indomethacin, bradykinin, endothelin-1, JZL 184, URB597 and AH6809 were supplied by Sigma Chemical Co. (UK). U46619, nimesulide, flurbiprofen, AM251, AM630, capsaicin, and L-161,982 were purchased from Tocris (UK). 2-AG was purchased from Ascent Scientific (UK). CAY10441 was purchased from Caymen Chemicals (Cambridge Bioscience, UK). l-NAME, indomethacin, nimesulide and flurbiprofen were dissolved in PSS. 2-AG, capsaicin, AH6809, CAY10441 and L-161,982 were dissolved in ethanol at 10 mmol/L with further dilutions made in distilled water. AM251, AM630, JZL184 and URB597 were dissolved in DMSO at 10 mmol/L with further dilutions made in distilled water. Bradykinin was dissolved in distilled water at 10 mmol/L.

## 3 Results

### 3.1 General

**Fig. 1:**
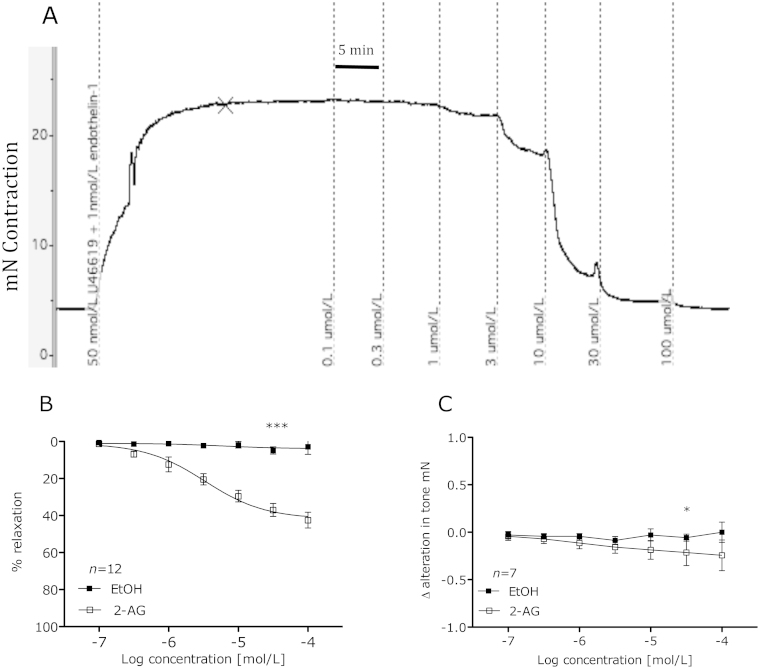
Trace data showing 2-AG-induced vasorelaxation (A). Vasorelaxant effects of 2-AG compared to vehicle-treated segments of mesenteric artery from the same patient (B). 2-AG effects on baseline tone compared to vehicle treated segments of mesenteric artery from same patient (C). Data given as means with error bars representing S.E.M. (Students paired *t-test* of area under curve *P < 0.05, ***P < 0.001).

Using wire myography, initial experiments revealed 2-AG caused concentration-dependant vasorelaxation of pre-constricted human mesenteric arteries with respective pEC_50_ and *R*_max_ values of 5.4 ± 0.2 and 42.4 ± 2.8 respectively (see Fig. 1A and B). 2-AG also caused a modest reduction in baseline tone (Fig. 1C). Subsequent investigations using a range of inhibitors and antagonists were then carried out to underpin the mechanisms of 2-AG-induced vasorelaxation.

### 3.2 Potential receptor and endothelium roles in 2-AG induced vasorelaxation

**Fig. 2:**
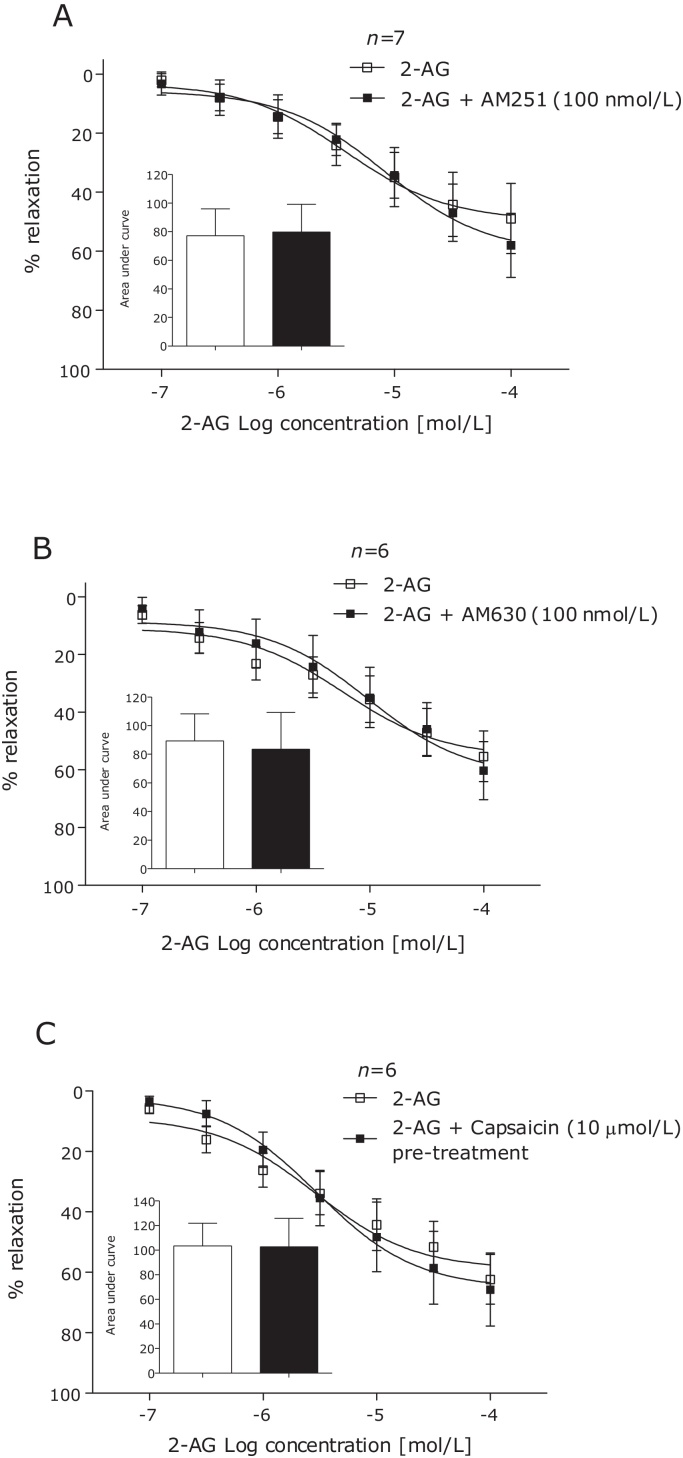
2-AG-induced vasorelaxation of human mesenteric arteries after 10 min incubation with the CB_1_ antagonist AM251 (100 nmol/L) (A), CB_2_ antagonist AM630 (100 nmol/L) (B) and after 1 h desensitisation of the TRPV1 receptor using the TRPV1 agonist capsaicin (10 μmol/L) (C). Data given as means with error bars representing S.E.M. Comparisons made between control and intervention segments of the same artery using Students paired *t-test* of area under curve.

**Fig. 3:**
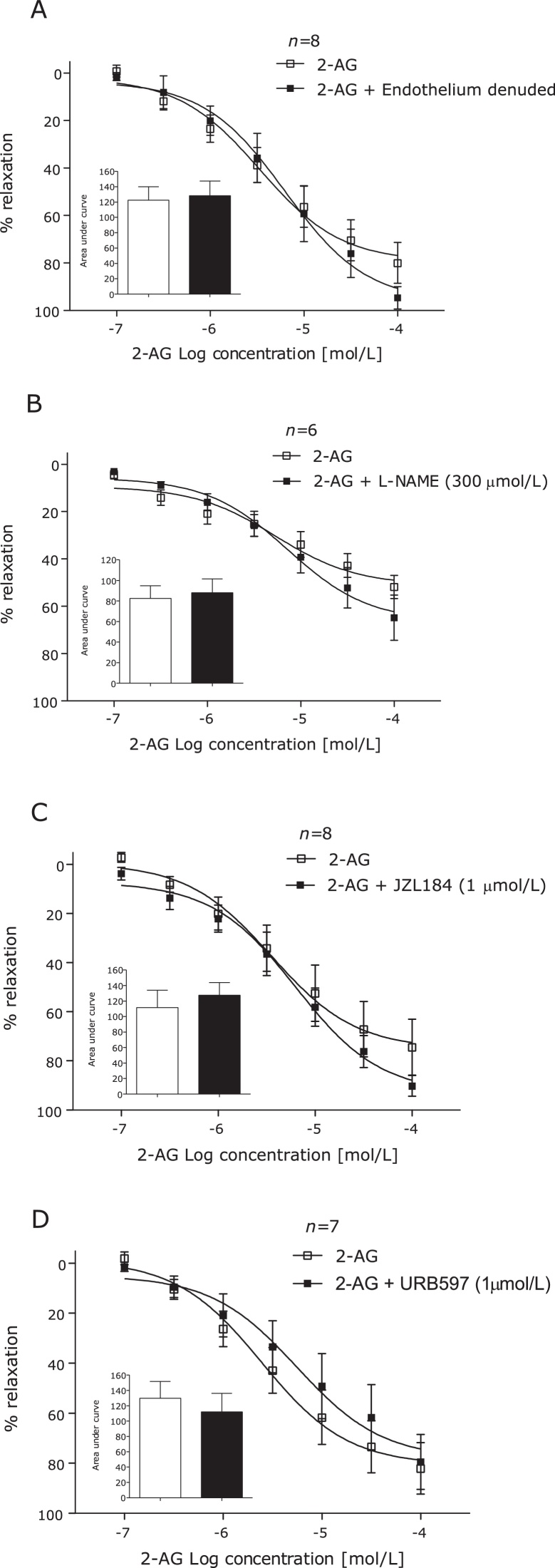
2-AG-induced vasorelaxation of human mesenteric arteries after removal of the endothelium (A), in the presence of l-NAME (300 μmol/L) (B), in the presence of the MAGL inhibitor JZL184 (1 μmol/L) (C) and the FAAH inhibitor URB597 (1 μmol/L). (D) Data given as means with error bars representing S.E.M. Comparisons made between control and intervention segments of the same artery using Students paired *t-test* of area under curve.

Antagonism of the CB_1_ (AM251, 100 nmol/L) or CB_2_ (AM630, 100 nmol/L) receptors (Fig. 2A and B), desensitisation of the TRPV1 receptor using capsaicin (10 μmol/L) (Fig. 2C), removal of the endothelium or incubation of endothelium intact arteries with l-NAME had no effect on vasorelaxation to 2-AG in human mesenteric arteries (Fig. 3A and B).

### 3.3 Potential roles for 2-AG metabolism in 2-AG induced vasorelaxation

**Fig. 4:**
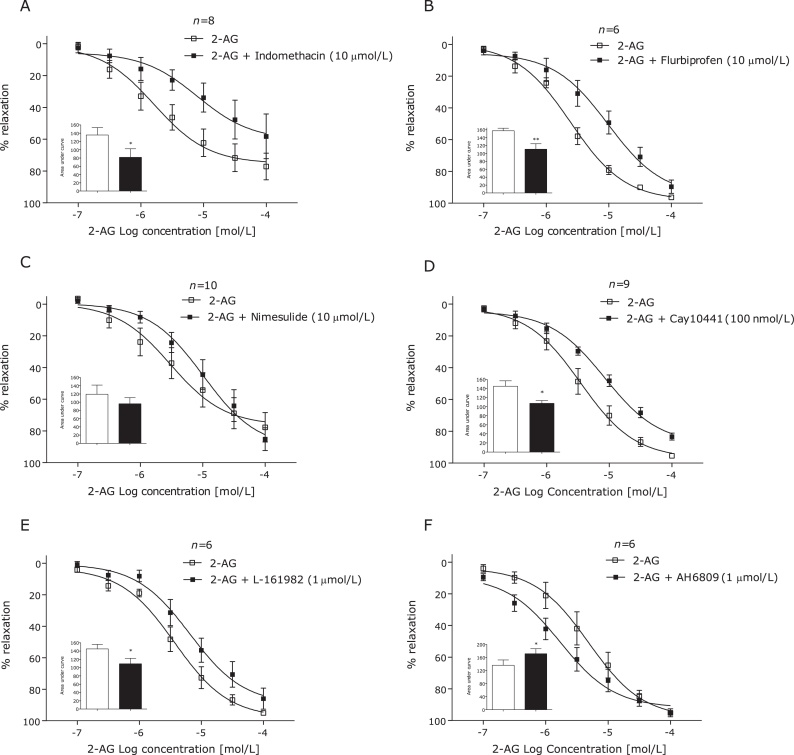
2-AG-induced vasorelaxation of human mesenteric arteries after addition of the non-selective COX inhibitor indomethacin (10 μmol/L) (A), the COX-1 inhibitor flurbiprofen (10 μmol/L) (B), the COX-2 inhibitor nimesulide (10 μmol/L) (C), the prostanoid IP receptor antagonist CAY10441 (100 nmol/L) (D) the prostanoid EP_4_ antagonist L-161,982 (1 mmol/L) (E) and the prostanoid EP_1_, EP_2_, EP_3_, DP and TP receptor antagonist (AH 6809, 1 μmol/L). Data given as means with error bars representing S.E.M. Comparisons made between control and intervention segments of the same artery using Students paired *t-test* of area under curve. **P < 0.01, ***P < 0.001.

Incubation with the MAGL inhibitor JZL184 (1 μmol/L) or the FAAH inhibitor URB597 (1 μmol/L) had no effect on 2-AG-induced vasorelaxation (Fig. 3C and D). However, incubation with the non-selective COX inhibitor indomethacin (10 μmol/L) significantly reduced 2-AG-induced vasorelaxation (Fig. 4A). Furthermore, the COX-1 inhibitor flurbiprofen (10 μmol/L), but not the COX-2 inhibitor nimesulide (10 μmol/L), inhibited 2-AG-induced vasorelaxation (Fig. 4B and C). Antagonism of the prostanoid IP (CAY10441, 100 nmol/L) and EP_4_ (L-161,982, 1 μmol/L) receptors significantly reduced the vasorelaxant responses to 2-AG (Fig. 4D and E). However, the prostanoid EP_1_, EP_2_, EP_3_, DP and TP receptor antagonist (AH 6809, 1 μmol/L) potentiated the response to 2-AG (Fig. 4D).

### 3.4 Potential ion channel involvement in 2-AG induced vasorelaxation

**Fig. 5:**
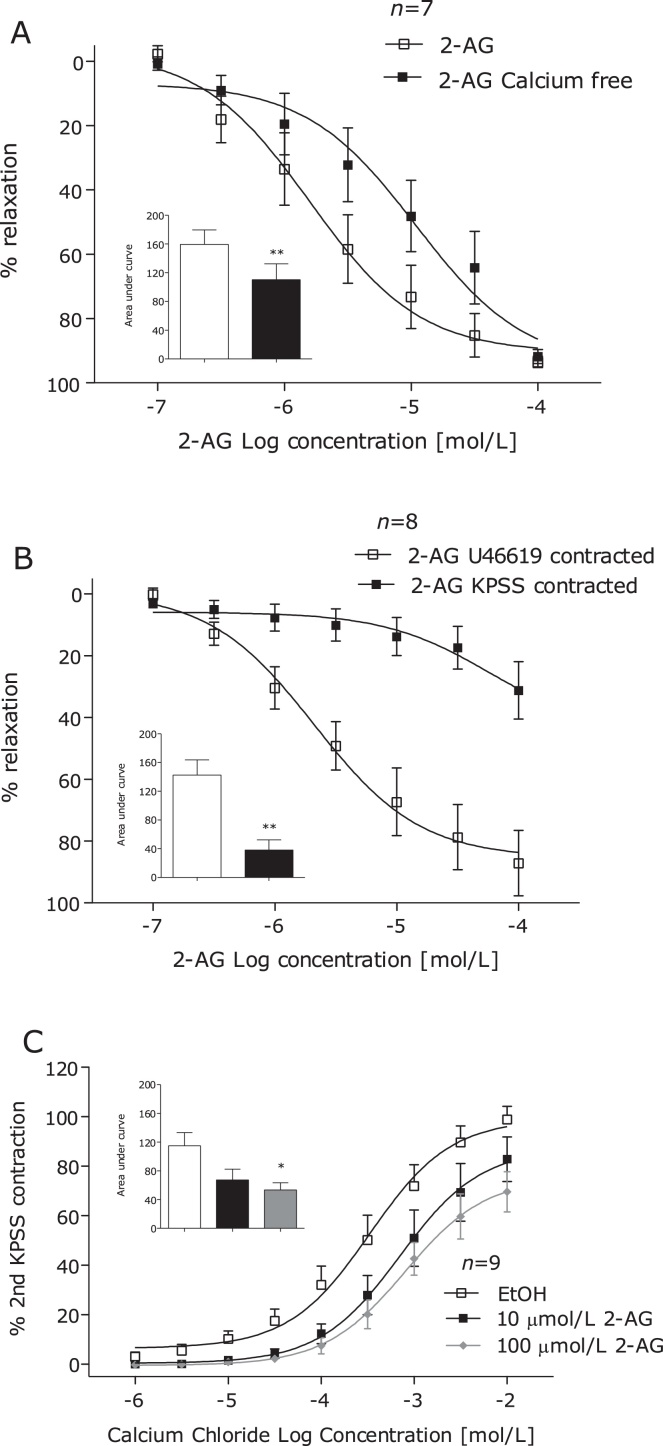
2-AG-induced vasorelaxation of human mesenteric arteries in arteries contracted with KPSS (124 mmol/L) (A) and U46619 in Ca^2+−^free PSS buffer (B). The effects of 10 min incubation with 2-AG (10 and 100 μmol/L) and vehicle control (0.1% EtOH) on CaCl_2_ contraction in Ca^2+^ free KPSS (124 mmol/L). Data given as means with error bars representing S.E.M. Comparisons made between control and intervention segments of the same artery using Students paired *t-test* of area under curve (A and B) and one way ANOVA of area under the curve with Dunnets *post hoc* test (C). *P < 0.05, **P < 0.01, ***P < 0.001.

In KPSS contracted arteries, 2-AG-induced vasorelaxation was inhibited (Fig. 5A). In arteries contracted with U46619 in calcium free PSS, 2-AG-induced vasorelaxation was significantly inhibited (Fig. 5B). In arteries bathed in high potassium calcium free solution and incubated with 2-AG, the maximum contraction to calcium chloride was inhibited in arteries incubated with 100 μmol/L, but not 10 μmol/L 2-AG (Fig. 5C).

### 3.5 The effect of patient co-morbidities on 2-AG-induced vasorelaxation

It should be noted that in patient numbers used in this study were too small to show conclusive data concerning the effects of various patient pathophysiology's on 2-AG responses. However, 2-AG pEC_50_ and/or *R*_max_ values often varied between patients. *Post hoc* analysis revealed that 2-AG pEC_50_ and/or *R*_max_ were reduced in those with heart disease, type two diabetes, those taking NSAID, statin and anti-diabetic medication (see supplementary data Table 5).

## 4 Discussion

There is growing interest in the role of 2-AG in cardiovascular disorders. This work is the first to characterise 2-AG-induced vasorelaxation in the human mesenteric artery, which is dependent on COX-1 metabolism, activation of the prostanoid EP_4_ and IP receptors, potassium hyperpolarisation and calcium channel modulation.

2-AG causes vasorelaxation in a variety vascular beds [6,11,8,9] and circulating 2-AG levels are increased in a range of CVS disorders [13–16]. However the direct effect of 2-AG on human arterial tone is unknown. We have shown that 2-AG relaxes human mesenteric arteries with a similar potency and efficacy to that observed in rat mesenteric arteries [9], but lower than that observed in rabbit mesenteric arteries [11]. The concentrations of 2-AG required to elicit vasorelaxation are above the reported physiological and pathophysiological plasma levels of 2-AG [1]. However, 2-AG is an unstable compound [36] that is produced on demand to function in a paracrine fashion [2]. Therefore, it is unlikely that plasma concentrations of 2-AG reflect cellular/local 2-AG concentrations.

Activation of CB_1_, CB_2_ or TRPV1 has been shown to partially underpin the vasoactive effects of other cannabinoid ligands [37], and 2-AG activation of the CB_1_ receptor causes vasorelaxation of rat cerebral arteries and rabbit mesenteric arteries [11,38]. The potential role of the CB_2_ and TRPV1 receptor in 2-AG induced vasorelaxation was previously untested. In the present study, 2-AG-induced vasorelaxation of the human mesenteric was not found to be mediated by CB_1_, CB_2_ and TRPV1 receptors, although all are present in human vascular smooth muscle cells [23,25,39] and endothelial cells [40,25]. The nuclear peroxisome proliferator activated receptors (PPAR) were also considered as potential target sites. Activation of the PPARγ receptor has been shown to increase cAMP [41], with both activation of PPARγ and increased cAMP leading to improved endothelial function and enhanced vasorelaxation in the aortae of diabetic mice [42]. Despite the findings that show 2-AG is a able to activate PPARγ receptors in human Jurkat cells [43] and that other endocannabinoids are able to cause vasorelaxation through PPARγ receptors [44] it seems unlikely that 2-AG is acting in through these pathways in the present study for several reasons. Firstly, in the present study 2-AG concentration response curves were completed in 45 min, however previous studies have shown that endocannabinoid/PPARγ mediated vasorelaxation is time-dependant with only slight effects occurring between 30 and 45 min [44]. Finally, cannabinoids have been shown to cause vasorelaxation through PPARγ in aortae but not mesenteric arteries [45]. Vasorelaxation to 2-AG and other endocannabinoids in animal models is endothelium-dependent [37,9]. However, we found that 2-AG-induced vasorelaxation in human mesenteric arteries is not inhibited by removal of the endothelium or inhibition of endogenous nitric oxide production. Furthermore, the maximal vasorelaxant response to 2-AG was not correlated with the endothelium-dependent response to bradykinin in the same patient. A lack of a role for the endothelium or nitric oxide production in the vascular response to 2-AG in human mesenteric arteries is in agreement with previous findings in rabbit mesenteric arteries [11], but not in rat mesenteric arteries [9] or bovine coronary arteries [8], possibly highlighting species differences in 2-AG vascular responses.

2-AG can be hydrolysed by MAGL and FAAH [3]. Hydrolysis of 2-AG, potentially through both these pathways has previously been shown to cause vasorelaxation of bovine coronary arteries [8]. However, we found that 2-AG-induced vasorelaxation in human mesenteric arteries is not mediated by 2-AG hydrolysis through MAGL or FAAH. COX-1 and -2 are expressed in human endothelium and smooth muscle cells [46], and 2-AG serves as a substrate for COX metabolism [47,48]. COX metabolism has been implicated in vasorelaxation to 2-AG in the bovine coronary artery [8], but not rat or rabbit mesenteric arteries [11,9]. We found that 2-AG-induced vasorelaxation of the human mesenteric artery is inhibited by the non-selective COX inhibitor indomethacin, the COX-1 favourable inhibitor flurbiprofen, but not the COX-2 inhibitor nimesulide. This suggests that 2-AG induced vasorelaxation occurs through COX-1 dependent pathways. However, previous works have shown that COX-1 metabolism of 2-AG is unlikely given that unlike COX-2, COX-1 does not express the Arg-513 residue that is key in the binding of 2-AG to COX-2. To inhibit COX-2, nimesulide was used in this study. Although interactions have been shown between nimesulide and Arg-513, Arg-513 is not reported as the main binding site of nimesulide to COX-2 [49–51]. Therefore, it is possible that 2-AG may have still had the ability to bind too, and be metabolised by COX-2 in the presence of nimesulide. However, the role for the metabolism of COX-1 in the vasorelaxant effects of 2-AG has previously been shown in the rat mesenteric artery where 2-AG-induced vasorelaxation was limited by COX-1 metabolism [9]. Taken with the present study, this would suggest a need for further research into the metabolism of 2-AG by both COX isoforms in the vasculature.

COX metabolism of 2-AG produces a range of novel prostanoid glycerol esters [52] that undergo hydrolysis to prostaglandins [53]. We therefore investigated potential roles for the DP_1_, EP_2_, EP_4_ and IP prostanoid receptors, which are all reported to cause vasorelaxation [54,55], and found that 2-AG-induced vasorelaxation is dependent on activation of the prostanoid EP_4_ and IP receptors. Similarly, in the rat aorta, the endocannabinoid anandamide causes vasorelaxation partly by activation of EP_4_ receptors [56]. Since, prostaglandin glycerol esters are unable to activate prostanoid receptors [52], it is likely that COX metabolism of 2-AG results in PGE_2_ and PGI_2_ formation, either directly or *via* prostaglandin glycerol ester production and subsequent hydrolysis to prostanoids. The finding that AH6809 potentiated 2-AG induced vasorelaxation might be explained due to AH6809 having affinity for prostanoid receptors that bring about vasoconstriction [57]. Metabolism of 2-AG through COX-1 may result in formation of both contractile and relaxant prostanoids, the balance of which is predominantly vasorelaxant.

When arteries were contracted using high potassium PSS to inhibit potassium efflux, 2-AG-induced vasorelaxation was greatly inhibited, suggesting that 2-AG-induced vasorelaxation is dependent on K^+^ channel activation. This is a finding common with a number of cannabinoid ligands in a range of vascular preparations [58,59] including human arteries [60]. To determine a potential role for calcium channels, some arteries were contracted using U46619 in calcium-free PSS, *i.e.* contraction dependent on release of calcium from intracellular stores. Under these conditions, partial inhibition of 2-AG-induced vasorelaxation was observed. Cannabinoids also cause vasorelaxation through inhibition of Ca^2+^ entry through voltage operated calcium channels [61]. In the present study, 2-AG, at the highest concentrations, inhibited the maximum contraction to calcium chloride. Taken together, these findings suggest that 2-AG modulates ion channel activation to favour vasorelaxation, which may be coupled to prostanoid receptor activation or a direct action.

The magnitude of vasorelaxation varied between patient samples, therefore *post hoc* analysis was carried out to determine if patient co-morbidities influenced the 2-AG response. These analyses revealed that 2-AG pEC_50_ and *R*_max_ responses were reduced in patients with heart disease, type two diabetes, those taking NSAID medication and those taking anti-diabetic medication. It must be noted that patient numbers used in this study were too small for adequate statistical analysis of these findings. However based on the findings of this study, future studies should investigate the effects of disease and medication on the endocannabinoid system in vascular function in humans.

## 5 Conclusion

We have shown for the first time that the endogenous cannabinoid 2-AG causes vasorelaxation of human mesenteric arteries. This was not mediated by CB_1_, CB_2_, TRPV1, the endothelium or metabolism by FAAH or MAGL. Rather, vasorelaxation to 2-AG in humans is dependent on COX-1 metabolism and subsequent activation of EP_4_ and IP prostanoid receptors. 2-AG responses vary greatly between patients, which may be related to underlying pathologies or medication. Further work is required to fully understand the role of 2-AG in human vasculature.

## Funding

This work was supported by the British Heart Foundation (FS/09/061).

## Conflict of interest

None declared.
