# Supplementary material for: Cyclooxygenase metabolism mediates vasorelaxation to 2-arachidonoylglycerol (2-AG) in human mesenteric arteries
Source: Pharmacol Res. 2014 Mar;81(100):74–82. doi: 10.1016/j.phrs.2014.02.001 (PMC3992009; doi:10.1016/j.phrs.2014.02.001)
Supplement: Supplementary file 1 [file mmc1.docx]

#

**

Table 1. Patient characteristics, diagnosis and medications. Samples were taken from 44 patients, but only 41 allowed research access to medical notes.

Table 2. Effects of a range of different treatments on 2-AG pEC_50_ and R_max_ values. Comparisons made using Students unpaired *t*-test. * *P*<0.05, ** *P*<0.01 and *** *P*<0.001.

Table 3. Effects of a range of inhibitors/antagonists on artery baseline tone and contractile responses. ∆ alteration in baseline tone determined by the baseline tone after addition of inhibitor/antagonist minus baseline tone before addition of inhibitor/antagonist. Contractile tone to U46619 (50-250 nmol/L) in combination with endothelin-1 (1-3 nmol/L) is expressed as percentage of response to 124 mmol/L KPSS. *n* is equal to the number of patients. Comparisons made using 1-way ANOVA with Dunnets *post-hoc* test to compare all responses to time control (***P*<0.01).

Table 4 Distribution of patients in between pharmacological protocols. Values expressed as percentage of total patients used for each individual protocol.

| Patient characteristic | Effects on 2-AG-induced vasorelaxation | | | | |
| --- | --- | --- | --- | --- | --- |
|  |  | Presenting characteristic | | Absence of characteristic | |
|  |  |  | |  | |
|  |  | mean ± s.e.m | *n* | mean ± s.e.m | *n* |
|  |  |  |  |  |  |
| Heart disease | pEC_50_ | 5.1±0.2 | 9 | 5.6±0.1 | 32 |
|  | R_max_ | 49.7±3.9 |  | 76.3±3.3*** |  |
|  |  |  |  |  |  |
| Hypercholesterolaemia | pEC_50_ | 5.4±0.2 | 16 | 5.6±0.1 | 25 |
|  | R_max_ | 65.4±5.0 |  | 75.5±3.8 |  |
|  |  |  |  |  |  |
| Type-2 diabetes | pEC_50_ | 5.4±0.3 | 10 | 5.5±0.1 | 31 |
|  | R_max_ | 58±7.1 |  | 76.3±3.0** |  |
|  |  |  |  |  |  |
| Hypertensive | pEC_50_ | 5.5±0.2 | 16 | 5.5±0.1 | 25 |
|  | R_max_ | 65±4.1 |  | 73±4.1 |  |
|  |  |  |  |  |  |
| BMI ≥25kg/m^2^ | pEC_50_ | 5.4±0.2 | 13 | 5.6±0.1 | 28 |
|  | R_max_ | 72.5±5.9 |  | 70.1±3.7 |  |
|  |  |  |  |  |  |
| Smoker | pEC_50_ | 5.2±0.2 | 11 | 5.6±0.1 | 30 |
|  | R_max_ | 78.0±6.3 |  | 68.8±3.5 |  |
|  |  |  |  |  |  |
| NSAID | pEC_50_ | 5.2±0.2 | 14 | 5.6±0.1* | 27 |
|  | R_max_ | 51.1±5.0 |  | 81.1±3.1*** |  |
|  |  |  |  |  |  |
| Statin | pEC_50_ | 5.3±0.2 | 16 | 5.6±0.1 | 25 |
|  | R_max_ | 57.6±5.0 |  | 77.4±3.3** |  |
|  |  |  |  |  |  |
| Hypoglycaemic medication | pEC_50_ | 5.1±0.3 | 6 | 5.5±0.1 | 35 |
|  | R_max_ | 43.2±5.8 |  | 74.8±3.0*** |  |
|  |  |  |  |  |  |
| Beta blocker | pEC_50_ | 5.3±0.2 | 8 | 5.6±0.1 | 33 |
|  | R_max_ | 65.1±4.8 |  | 69.9±3.5 |  |
|  |  |  |  |  |  |
| ACE inhibitor | pEC_50_ | 5.5±0.3 | 6 | 5.5±0.1 | 35 |
|  | R_max_ | 65.4±6.8 |  | 70.7±3.3 |  |

Table 5. Effects of a range of co-morbidities on 2-AG pEC_50_ and R_max_ values. comparisons made between patients diagnosed/prescribed with a characteristic/medication and those without using Students unpaired *t*-test. * *P*<0.05, ** *P*<0.01 and *** *P*<0.001.

**

Figure 1. (A) 124 mmol/L KPSS responses in arteries used fresh or after overnight storage. (B) 50 nmol/L U46619 responses expressed as a percentage of 124 nmol/LKPSS response in arteries used fresh or after overnight storage. (C) 10 μmol/L bradykinin responses in arteries used fresh or after overnight storage. (D) 2-AG and ethanol vehicle response curves in arteries used fresh or after overnight storage. (E) 2-AG responses in arteries taken from patients undergoing different operations (Right hemicolectomy, Left hemicolectomy, Sigmoid colectomy, AR: Anterior resection/ abdominoperineal resection, Total colectomy). Data given as means with error bars representing S.E.M. Comparisons made using Students' unpaired *t*-test (A-C) or Students unpaired *t‑*test of area under the curve (D) or 1-way ANOVA with Bonferroni *post-hoc test* of area under the curve (E).

Figure 2. (A) 2-AG concentration response curves in males compared to females, comparisons made using Students unpaired *t-*test of area under the curve. (B) 100 μmol/L 2-AG responses correlated with age using linear regression. (C) 10 μmol/L bradykinin responses compared between males and females, comparisons made using Students unpaired *t-*test. (D) 10 μmol/L bradykinin responses correlated with patient age using linear regression.
